# Using Medical Emergency Teams to detect preventable adverse events

**DOI:** 10.1186/cc7983

**Published:** 2009-07-30

**Authors:** Akshai Iyengar, Alan Baxter, Alan J Forster

**Affiliations:** 1 Department of Medicine, Faculty of Medicine, University of Ottawa, 451 Smyth Road, Ottawa, ON, K1H 8M5, Canada; 2 Department of Anaesthesia, Faculty of Medicine, University of Ottawa, 451 Smyth Road, Ottawa, ON, K1H 8M5, Canada; 3 Clinical Epidemiology Program, Ottawa Hospital Research Institute, 725 Parkdale Avenue, Ottawa, ON, K1Y 4E9, Canada

## Abstract

**Introduction:**

Medical Emergency Teams (METs), also known as Rapid Response Teams, are recommended as a patient safety measure. A potential benefit of implementing an MET is the capacity to systematically assess preventable adverse events, which are defined as poor outcomes caused by errors or system design flaws. We describe how we used MET calls to systematically identify preventable adverse events in an academic tertiary care hospital, and describe our surveillance results.

**Methods:**

For four weeks we collected standard information on consecutive MET calls. Within a week of the MET call, a multi-disciplinary team reviewed the information and rated the cause of the outcome using a previously developed rating scale. We classified the type and severity of the preventable adverse event.

**Results:**

We captured information on all 65 MET calls occurring during the study period. Of these, 16 (24%, 95% confidence interval [CI] 16%–36%) were felt to be preventable adverse events. The most common cause of the preventable adverse events was error in providing appropriate therapy despite an accurate diagnosis. One service accounted for a disproportionate number of preventable adverse events (n = 5, [31%, 95% CI 14%–56%]).

**Conclusions:**

Our method of reviewing MET calls was easy to implement and yielded important results. Hospitals maintaining an MET can use our method as a preventable adverse event detection system at little additional cost.

## Introduction

Medical Emergency Teams (METs), alternatively known as Rapid Response Teams, have recently been implemented in many hospitals worldwide [1]. The primary role of an MET is to improve the early identification and management of acutely deteriorating ward patients [1]. Several studies demonstrate an association between MET implementation and improved hospital outcomes [2-5], although there are also negative trials [6-8]. Despite the conflicting evidence, many institutions and health systems have continued to fund MET implementations due to perceived benefits extending beyond those evaluated in the published research [9-11]. These include improvements in patient safety culture and nursing work environment.

In this study, we report on our experience with expanding the role of our institution's MET to support the detection of preventable adverse events, which are defined as poor outcomes caused by medical error. We felt a systematic evaluation of patient care immediately prior to MET notification might provide useful information for system improvement because the MET is responding to critical situations in which there is at least some likelihood of prior inappropriate treatment [12-16]. Our method is a modification of a prior attempt to achieve a similar objective [17]. Our approach differs in that we wished to incorporate the evaluation as part of the routine followed by the MET during a call. We hoped that this would minimize the resources required for the task and enhance timeliness of our detection while at the same time yield useful information.

## Materials and methods

### Setting

The study was approved by the Ottawa Hospital Research Ethics Board. The Ottawa Hospital General Campus is a 487-bed tertiary care teaching hospital. It implemented an MET in January 2005. The team is composed of a physician (intensivist during the day and a critical care resident at night), a critical care nurse, and a critical care-trained respiratory therapist. The MET can be activated by any hospital staff and is active 24 hours a day. Providers in our hospital use standard criteria for activating the MET. The MET has over 40 calls per 1,000 hospital admissions, and more than 70% of intensive care unit admissions are preceded by an MET call.

### Data collection

For a 4-week period in 2007, we used a standard form to collect information on each MET call (Appendix 1 of Additional data file 1). For each MET call, we described the reason for the call, the admitting service and diagnosis, the admission status, the current acute and chronic medical conditions, a summary of the patient's hospital stay and course in hospital, the presumed explanation for the patient's deterioration, the treatment provided by the attending team prior to the MET call, the MET's treatment, and the patient's eventual outcome. The MET physician recorded data at the time of the MET call Monday through Friday during working hours. For MET calls at other times, the MET physician interviewed the providers involved in the case and reviewed the medical record. It took approximately 5 minutes to complete the form.

### Outcomes

We used standard patient safety definitions [18]. An *adverse outcome *is any suboptimal outcome experienced by the patient. By definition, any MET call is an adverse outcome. An *adverse event *is an adverse outcome caused by the processes of medical management rather than by the progression of disease. Medical management refers to all aspects of care. A *preventable adverse event *is an adverse event caused by error or health system flaw. An error is a failure to achieve a desired objective through the failure to execute a plan correctly, through the implementation of an incorrect plan, or through omission.

### Case classification

All cases were reviewed and classified by three physicians – an internist (AJF), an anesthetist/intensivist (AB), and a PGY2 (post-graduate year two) internal medicine resident (AI) – within 1 week of each MET call. The group of three physicians achieved consensus on whether the outcome was a result of medical management using a previously derived and widely accepted review process [19-24]. If so, the case was considered an adverse event, in which case it was further classified in terms of its preventability. Preventable adverse events were further classified as to their subtype.

### Consent

We did not obtain patient or provider consent as part of the protocol. We argued successfully to our Research Ethics Board that the protocol posed minimal risk to patients or providers. The principal ethical concern was the potential of an inappropriate disclosure of personal health information. We created a case report form that did not contain usual patient or provider identifiers. Individuals could be identified only if someone obtained our case report forms and used our hospital information systems inappropriately.

### Statistical analysis

We created descriptive statistics for all studied factors. We compared the distribution of these factors by preventable adverse event status using the chi-square statistic for categorical variables and the Wilcoxon rank-sum test for continuous variables. As only one variable was significantly associated with adverse event status, we did not perform a multi-variable analysis. We used SAS version 9.1 (SAS Institute Inc., Cary, NC, USA) for all analyses.

## Results

Sixty-five MET calls occurred during the study period (Table 1). Patients were elderly (median age 71 years, interquartile range 60 to 82 years). Most hospital services had at least one MET call. Ninety-one percent of patients were considered 'acute care' at the time of the MET call and had been in hospital for a median of 4 days (interquartile range 2 to 12.5 days) before the call. Of the 65 calls received, 23 were considered to be adverse events (35%, 95% confidence interval [CI] 25% to 48%) and 16 were considered to be preventable adverse events (24%, 95% CI 16% to 36%). Calls of three of the sixteen patients with preventable adverse events were considered life-threatening (19%, 95% CI 7% to 43%). Six MET cases and their ratings are described as examples in the text box of Additional data file 2. We describe all adverse events in Appendix 2 of Additional data file 3.

**Table 1:** Characteristics of Medical Emergency Team calls

Characteristic	All	Patients with preventable AEs	Patients without preventable AEs	*P *value
Number	65	16	49	N/A
Age, years	71 (60–81)	76 (68–82)	68 (59–81)	0.32
Service				0.02
A	12 (18%)	1 (6%)	11 (22%)	
B	8 (12%)	2 (13%)	6 (12%)	
C	7 (11%)	5 (31%)	2 (4%)	
D	5 (8%)	2 (13%)	3 (6%)	
E	5 (8%)	1 (6%)	4 (8%)	
F	4 (6%)	2 (13%)	2 (4%)	
Other	24 (37%)	3 (19%)	21 (43%)	
Admission status				0.31
Acute	59 (91%)	14 (88%)	45 (92%)	
Chronic	6 (9%)	2 (13%)	4 (8%)	
Length of stay, days^a^	4 (2–12.5)	4 (3–21)	4 (2–11)	0.51
Call indication				0.23
Blood pressure	23 (35%)	5 (31%)	18 (37%)	
Airway	11 (17%)	1 (6%)	10 (20%)	
Heart rate	8 (12%)	3 (19%)	5 (10%)	
Oxygen saturation	9 (14%)	3 (19%)	6 (12%)	
Respiratory rate	2 (3%)	1 (6%)	1 (2%)	
Urine output	1 (2%)	1 (6%)	0	
Other	11 (17%)	2 (13%)	9 (18%)	
Time of day^b^				
Day (8 a.m.-5 p.m.)	32 (49%)	6 (38%)	26 (53%)	
Night (5 p.m.-8 a.m.)	33 (51%)	10 (63%)	23 (47%)	

Values other than *P *value and number of patients are presented as median (range) or as number (percentage). *P *value represents the probability of an error when concluding that the characteristic differs by adverse event (AE) status. ^a^Length of stay in hospital before Medical Emergency Team (MET) call; ^b^time of day of MET call. N/A, not applicable.

'Therapeutic errors', defined as a failure to apply the appropriate treatment regimen, contributed to the outcome in 14 of the 16 patients with preventable adverse events (88%, 95% CI 64% to 97%). The other two preventable adverse events were considered adverse drug events.

We assessed factors associated with preventable adverse event classifications (Table 1). The only characteristic associated with preventable adverse event occurrence was hospital service. Service C was noted to have a high proportion of calls related to preventable adverse events. Although service A accounted for the most calls, it accounted for only one preventable adverse event. All other studied factors were not associated with preventable adverse event status.

## Discussion

We found our MET-based approach for preventable adverse event detection to be simple to implement, easy to maintain, and informative for quality improvement efforts. One quarter of MET calls were associated with preventable adverse events. We found one service responsible for a disproportionate number of preventable adverse events. We also found inappropriate responses to critical patients as the most common cause of preventable adverse events. Our hospital is using this information to guide quality improvement strategies.

Our program cost very little to implement. Although we collected data specifically for the study, it is possible for the care providers present at the MET call to incorporate information collected at each call directly into the routine. The program required weekly meetings, which lasted less than an hour and could be performed remotely using telephone conferencing. This task was not onerous for the physicians participating in the program and was seen as part of their professional obligations of monitoring the effectiveness of the hospital system.

Although we believe our methodology is easily replicable, our surveillance results should not be generalized. Our study was performed in a single site for a limited period of time. Despite the relatively short observation period, we did identify a statistically significant and clinically plausible pattern of factors associated with preventable adverse events. Prior research has suggested that, even in acute care hospitals, there is often an inadequate response to critically ill patients [12-16]. Furthermore, a prior program similar to ours, but which observed care for 8 months, found a similar proportion of MET calls to be related to preventable adverse events [17]. In the prior study, the predominant problem was diagnostic error. It is possible that, if the observation period of our study had been longer, we would have found different patterns. It is also possible that our studies used slightly different terminologies to classify the type of adverse events. Thus, despite the consistency with prior research, we recommend a larger study. Such a study should ensure standard terminology and consider comparing the preventable adverse events detected by this method with those identified using other methods to ensure validation of the types of preventable adverse events occurring in an institution.

Similarly, it is important to consider specific biases inherent in this approach to finding care-related problems in a hospital. The physician review process is biased by knowledge of outcome severity and by our natural and variable inclinations to find fault [25,26]. These biases can be minimized by having multiple reviewers [27] and by blinding outcome severity [25]. However, the impact of these biases can be mitigated but not entirely removed. As a result, any findings from an MET-based surveillance program should be interpreted and communicated cautiously. We suggest that they function as a starting point for assessments that are more intensive rather than as the basis of sanctions. Furthermore, we strongly suggest adopting a communication strategy that avoids blaming individuals or groups for negligence or incompetence. Rather, the findings should be used in a constructive and collaborative manner to plan future assessments and quality improvement efforts.

## Conclusions

Given the widespread implementation of METs, our proposed approach could immediately offer many hospitals an efficient method for monitoring preventable adverse events. This is an important advance given the apparent widespread patient safety problems in hospitals [19,20,28-30] and the inadequacy of existing surveillance systems [31-33].

## Key messages

• Medical emergency teams (METs) provide care to critically ill ward-based inpatients. METs have been implemented in many hospitals worldwide.

• METs often respond to clinical events in which there has been inappropriate antecedent care. Therefore, METs could form the basis of a preventable adverse event detection system.

• We have adopted a structured method of data collection and peer review to be used by METs to assist institutional learning regarding the avoidance of preventable adverse events.

• We have determined our method to be feasible.

• We have demonstrated the method's capacity to document important quality issues in the care of critically ill patients.

## Abbreviations

CI: confidence interval; MET: Medical Emergency Team.

## Competing interests

The authors declare that they have no competing interests.

## Authors' contributions

AJF conceived of the idea of the study and helped to facilitate data collection and provide important intellectual contributions during preparation of the manuscript. AI and AB helped to facilitate data collection and provide important intellectual contributions during preparation of the manuscript. All authors read and approved the final manuscript.

## Supplementary Material

Additional file 1 Appendix 1 containing our case review form. Click here for file

Additional file 2 A text box with several examples of adverse events identified during the study. Click here for file

Additional file 3 Appendix 2 with descriptions of all adverse events identified during the study. Click here for file
