# Supplementary material for: Using Medical Emergency Teams to detect preventable adverse events
Source: Crit Care. 2009 Jul 30;13(4):R126. doi: 10.1186/cc7983 (PMC2750180; doi:10.1186/cc7983)
Supplement: Additional file 1 — Appendix 1 containing our case review form. [file cc7983-S1.DOC]

Appendix 1 - **RACE Call Description and Rating Form**

**EVENT DETAILS**

MRN:11111111 Unit: 5nw Date and Time of call: 15oct07 Event no.:1

**OUTCOME DESCRIPTION *(To be completed by RACE team member)***

1. ***Describe the outcome and contextual details***

Context: Age; gender; admitting service; admitting diagnosis; important comorbidities; Length of stay in hospital before event;

Outcome: What prompted the RACE call; what was going on in the hours/days leading up to the RACE call

1. ***What was the response to the problem by the healthcare team prior to the RACE team arrival?***
2. ***What was the RACE team’s response to the problem?***
3. ***What was the documented cause of the problem?***
4. ***How severe was the outcome?***

***a***) Temporary physiological derangements; b) Permanent disability; c) Death

--this may not be known at the time of rating but we should track it down after the fact

**RATINGS *(To be completed by group consensus)***

1. Please rate the degree to which the outcome or its severity was influenced by medical management?

a. It was entirely due to the patients underlying condition

b. It was most likely due to the patients underlying condition

c. It was a close call but more likely due to the underlying condition

d. It was a close call but more likely due to the medical management

e. It was most likely due to the medical management

f. It was definitely due to the medical management

1. Was the outcome caused by an error (if the outcome or its severity was more likely due to medical management?

a. Definitely not due to error

b. More than likely not due to error

c. It was a close call but more likely not due to error

d. It was a close call but more likely due to error

e. More than likely due to an error

f. Definitely due to an error
