# Supplementary material for: Using Medical Emergency Teams to detect preventable adverse events
Source: Crit Care. 2009 Jul 30;13(4):R126. doi: 10.1186/cc7983 (PMC2750180; doi:10.1186/cc7983)
Supplement: Additional file 2 — A text box with several examples of adverse events identified during the study. [file cc7983-S2.DOC]

**Text Box: Examples of cases and ratings**

x

**Case 1** – Patient had acute distress related to acute pulmonary emboli in the context of malignancy despite DVT prophylaxis. *Not an adverse event*.

**Case 2** – Patient had upper gastrointestinal bleeding secondary to gastric malignancy leading to upper airway compromise and hypotension. *Not an adverse event*.

**Case 3** – Patient admitted for elective surgery and experienced hypersensitivity to spinal anaesthesia resulting in respiratory compromise. *Adverse event, non-preventable.*

**Case 4** - Pulmonary patient experienced syncope related to flouroquinolone related QTc prolongation. *Adverse event, non-preventable.*

**Case 5** – Elderly patient with metastatic cancer. Patient developed decreased level of consciousness due to inappropriate high dose of narcotic *Preventable adverse event.*

**Case 6** – Despite recognizing a diagnosis in a patient with an effective treatment, there was a several hour delay in administering appropriate therapy. As a result the patient experienced respiratory compromise. *Preventable adverse event.*
