# Supplementary material for: Using Medical Emergency Teams to detect preventable adverse events
Source: Crit Care. 2009 Jul 30;13(4):R126. doi: 10.1186/cc7983 (PMC2750180; doi:10.1186/cc7983)
Supplement: Additional file 3 — Appendix 2 with descriptions of all adverse events identified during the study. [file cc7983-S3.DOC]

**Appendix 2**

| **ID** | **Case Description** | **Type** | **Error** |
| --- | --- | --- | --- |
| 1 | Elderly patient admitted for emergent surgery. Post operative hypotension due to hypovolemia. Inappropriate fluid resuscitation. | Therapeutic | 1 |
| 2 | Elderly patient with metastatic cancer. Decreased level of consciousness due to inappropriate high dose of narcotic. | ADE | 1 |
| 3 | Elderly patient with metastatic cancer. Decreased level of consciousness due to inappropriate high dose of narcotic. | ADE | 1 |
| 4 | Elderly patient admitted for emergent surgery. Patient’s antihypertensive medications were not ordered. The patient experienced a hypertensive emergency. | Therapeutic | 1 |
| 5 | Elderly patient admitted for emergent surgery. Inappropriate response to hypotension. | Therapeutic | 1 |
| 6 | Elderly patient with metastatic cancer. Inappropriate response to hypotension. | Therapeutic | 1 |
| 7 | Elderly patient with stroke awaiting placement in a long term care facility. Inappropriate response to hypotension. | Therapeutic | 1 |
| 8 | Elderly patient with complex medical disease admitted with complication of surgery. Patient developed heart failure as a result of standard intravenous fluid therapy. | Therapeutic | 0 |
| 9 | Elderly patient admitted for emergent surgery. Patient experienced bradycardia and hypotension as a result of a prescribing error related to a beta blocker. | Therapeutic | 1 |
| 10 | Patient admitted for elective surgery and experienced hypersensitivity to spinal anaesthesia resulting in respiratory compromise. | Procedural | 0 |
| 11 | Patient developed respiratory compromise to an appropriately dosed and administered narcotic, which was prescribed for post-procedure analgesia. | ADE | 0 |
| 12 | Pulmonary patient experienced syncope related to flouroquine related QTc prolongation. | ADE | 0 |
| 13 | Patient experiences profound hypotension shortly following an elective procedure. | Therapeutic | 0 |
| 14 | Patient with metastatic cancer who receives excessive intravenous fluids and as a result experiences pulmonary edema. | Therapeutic | 1 |
| 15 | Elderly patient admitted for emergent surgery. Patient’s antihypertensive medications were not ordered. The patient experienced a hypertensive emergency | Therapeutic | 1 |
| 16 | Patient bled from NSAID induced gastric ulcers. | ADE | 0 |
| 17 | Post-operative patient experienced tachycardia due to inappropriate decision to discontinue diltiazem in a patient with atrial fibrillation. | Therapeutic | 1 |
| 18 | Volume management in elderly patient with complex medical conditions was inappropriate. This led to volume overload and pulmonary edema. | Therapeutic | 1 |
| 19 | Elderly patient with complex medical condition developed hypotension. Response to critical situation was inadequate and patient suffered adverse consequences as a result. | Therapeutic | 1 |
| 20 | Despite recognizing a diagnosis in a patient with an effective treatment, there was a several hour delay in administering appropriate therapy. As a result the patient experienced respiratory compromise. | Therapeutic | 1 |
| 21 | Syncope in patient following a thoracentesis with removal of 1 liter pleural fluid. | Therapeutic | 0 |
| 22 | Inappropriate management of patient with an acute coronary syndrome led to delay in definitive treatment. | Therapeutic | 1 |
| 23 | Patient receiving chemotherapy did not receive appropriate pre-medication regimen. As a result, the patient experienced rigours and hypotension. | Therapeutic | 1 |

NSAID=non-steroidal anti-inflammatory drug; Error=Case rated as a preventable adverse event, 1=yes, 0=no
